# What is a normal left ventricular ejection fraction in healthy adults? A meta-analysis of population-based echocardiographic studies

**DOI:** 10.1186/s44348-025-00063-4

**Published:** 2026-01-22

**Authors:** Anne Emilie Morsing, Filip Gnesin, Asya Lyass, Charlotte Andersson

**Affiliations:** 1 Graduate School of Health, Aarhus University, Aarhus, Denmark; 2 Department of Cardiology, Nordsjællands Hospital, Hillerød, Denmark; 3 Center for Advanced Heart Disease, Brigham and Women’s Hospital, Boston, MA USA; 4 Department of Mathematics and Statistics, Boston University, Boston, MA USA; 5 Department of Cardiology, Herlev and Gentofte Hospital, Herlev, Denmark

**Keywords:** Left ventricular ejection fraction, Normal value, Demography

## Abstract

**Background:**

Transthoracic echocardiography derived left ventricular ejection fraction (LVEF) is a cornerstone in heart failure risk prevention. However, the lower limits of normal LVEF remains imprecisely defined. We aimed to define normal LVEF ranges by sex, age group, and self-reported race/ethnicity using data from population-based echocardiographic studies.

**Methods:**

We systematically searched MEDLINE for studies published between January 1, 2000, and January 3, 2025, that reported the mean and standard deviation of LVEF measured by 2D or 3D echocardiography in healthy, community-based adult populations.

**Results:**

In 10 studies (n = 10,427; female sex, 48%), the pooled mean LVEF was 62.8% (95% confidence interval, 61.0%–64.7%), with estimated lower and upper normal limits of 51.8% and 73.2%, respectively. Women had higher mean LVEF (63.7%) than men (61.9%), with corresponding lower normal limits of 52.7% and 51.7%, respectively. LVEF was similar across age groups. Individuals of Asian origin had 2 to 3 percentage points higher LVEF than Black or White individuals, with lower normal limits of 54% for women and 53% for men. Fewer than 1% of women and approximately 1% of men would be expected to have an LVEF below 50%. Across all demographic subgroups, the probability that an LVEF < 50% is within the normal range was < 5%. There was significant heterogeneity of the included studies (e.g., τ^2^ = 8.82, I^2^ = 99.7% for overall analysis) that appeared unexplained by sex, age, or echocardiography modality (2D vs. 3D).

**Conclusions:**

In healthy adults, the lower limit of normal LVEF is approximately 53% for women and 52% for men, with slightly higher thresholds among individuals of Asian origin. An LVEF < 50% is highly unlikely to reflect normal function, regardless of sex, age, or self-reported race/ethnicity. Given the high statistical heterogeneity, the results should be interpreted with caution.

**Supplementary Information:**

The online version contains supplementary material available at 10.1186/s44348-025-00063-4.

## Background

Dilated cardiomyopathy and heart failure exhibit subclinical myocardial changes years prior to patients presenting with symptoms, offering a window of opportunity for early, disease modifying therapy. However, to establish whom should be treated, well-defined thresholds for defining abnormal left ventricular ejection fraction (LVEF) are needed. The most contemporary and widely adopted 2015 American Society of Echocardiography (ASE) and European Association of Cardiovascular Imaging (EACI) guidelines state that an LVEF < 52% for men and < 54% for women are suggestive of abnormal systolic function [1].

These data were derived from selected populations of mostly White individuals (Asklepios [2], Cardia [3], Flemehgho [4], and Padua [5]). The World Alliance Society of Echocardiography (WASE) Normal Values Study initiative has more recently suggested that LVEF may vary by race and that a normal LVEF is substantially higher than previously recognized by the guidelines (i.e., 58% for women and 57% for men) [6]. However, in a large, real-world cohort of patients undergoing transthoracic echocardiograms the long-term mortality appeared to be identical for those defined as normal by only the ASE/EACI guidelines compared with those who were normal by both WASE and ASE/EACI, suggesting that WASE might overclassify individuals as abnormal [7].

Defining normal ranges of LVEF is essential to guide preventive strategies while avoiding unnecessary downstream testing and treatment among individuals who are, in fact, healthy. In this meta-analysis, we aimed to establish updated, demographically sensitive reference ranges for LVEF using data from population-based cohorts across sex, age, and self-reported race/ethnicity.

## Methods

### Search strategy

We conducted a systematic review and meta-analysis in accordance with PRISMA (Preferred Reporting Items for Systematic reviews and Meta-Analyses) guidelines [8]. Two independent reviewers (AEM and CA) searched PubMed based on predefined search strings (Supplementary Table 1). The search was completed on January 3, 2025. Titles, abstracts, and full texts were screened independently by the two reviewers. Disagreements were resolved by consensus with a third investigator (FG). A total of 1,776 articles were screened, and 10 studies met inclusion criteria for the final analysis (Supplementary Fig. 1).

### Eligibility criteria

We included original studies published in or after 2000 that reported sex-specific mean and standard deviation (SD) of LVEF in healthy adult populations (≥ 18 years) assessed by 2D or 3D transthoracic echocardiography. Only articles published in English were eligible. Studies of pediatric populations, as well as those that did not provide sufficient statistical data (number of subjects, mean ± SD or enough detail to calculate SD), were excluded. We did not consider the control groups of case–control series of various disease states due to a risk of biased estimates (oversampling in certain age groups, ill-defined control groups, etc.). Similar, we excluded clinical samples of patients referred to echocardiogram on the suspicion for disease, even if found to have no overt cardiac disease by echocardiograms due to concerns for residual disorders that could influence LVEF estimates (e.g., anemia, hyperthyroidism, malignancy).

Studies using alternative imaging modalities such as cardiac magnetic resonance imaging or nuclear imaging (e.g., single-photon emission computed tomography) were excluded to reduce methodological heterogeneity, as echocardiography is the most widely used and accessible modality in routine clinical practice. Reviews, non-English publications, and literature published before 2000 were excluded.

### Data extraction

After study inclusion was finalized, one investigator (AEM) extracted the following data from each article: first author and year of publication, country, population characteristics, sample size, percentage of female participants, mean age, echocardiographic method (2D vs. 3D), equipment used and reported LVEF. When both 2D and 3D LVEF values were reported, 2D values were preferentially included to reflect common clinical practice and ensure conservative estimates, given their greater measurement variability. Definitions of a healthy adult varied across studies; specific inclusion and exclusion criteria for each are provided in Supplementary Table 2.

### Statistical analysis

LVEF was assumed to follow a normal distribution based on prior literature and the characteristics of population-based samples [9]. Each study’s mean LVEF and variance (SD^2^) was used to conduct random-effects meta-analyses (restricted maximum likelihood). Pooled mean estimates were computed directly, while pooled SDs were obtained by meta-analyzing log-transformed variance estimates and then back-transformed. Between-study heterogeneity was assessed using the τ^2^ and I^2^ statistic. Analyses were conducted in the overall sample, as well as stratified by sex, race, and age groups.

Reference limits for normal LVEF were defined as the pooled mean ± 1.96 × (pooled SD), corresponding to the central 95% of a normally distributed population. To evaluate whether specific LVEF values fell within the normal range, z-scores were calculated for LVEF values from 55% down to 45% in 1% decrements. One-sided P-values were derived by comparing each z-score against the lower 2.5th percentile of the normal distribution. Age effects were examined using meta-regression, modeling age as a categorical variable and stratifying by sex. Tests for differences across age strata were performed using analysis of variance test. All analyses were performed using R (R Foundation for Statistical Computing). Alpha level of 0.05 was considered statistically significant for all analyses.

## Results

### Study selection and characteristics

Ten studies comprising a total of 10,427 healthy adults who underwent either 2D or 3D echocardiography were included in the final analysis. Study and participant characteristics are summarized in Table 1 [10–19].

**Table 1:** Baseline characteristics

Study	Country	Population	No. of subjects	Female sex (%)	Age (yr)^a^			Echocardiographic method	Equipment
					All	Women	Men		
Addetia et al. [14] (2022)	USA, Canada, Mexico, Brazil, Argentina, Australia, Nigeria, India, China, Japan, Republic of Korea, Philippines, Iran, Italy, UK	WASE normal value study	1,589	47.6	47 ± 17	47 ± 17	48 ± 17	3D	GE, Philips, Siemens^b^
Aune et al. [15] (2010)	Norway	Current or former hospital staff	166	52.4	-	-	-	3D	Philips iE33
Bhambhani et al. [16] (2018)	India	Singel-center prospective study	133	44.4	39 ± 13	39 ± 15	39 ± 12	3D	Philips iE33
Chahal et al. [13] (2012)	UK	LOLIPOP	978	49.0	54 ± 10	European women: 55 ± 10 Indian women: 53 ± 10	European men: 54 ± 9 Indian men: 52 ± 10	Simpson 2D and 3D	Philips iE33
Eriksen-Volnes et al. [17] (2023)	Norway	HUNT4	1,412	55.8	-	57 ± 12	58 ± 12	Simpson 2D and 3D	GE Vivid E95
Mukherjee et al. [12] (2021)	India	Cross-sectional observational, single hospital study	1,377	43.9	27 ± 6	26 ± 6	27 ± 6	Simpson 2D	GE Vivid S5
Nel et al. [11] (2020)	South Africa	Prospective single-center study at Chris Hani Baragwanath Academic Hospital	253	59.3	36 ± 12	37 ± 13	35 ± 12	Simpson 2D	Phillips iE33
Rashid et al. [18] (2023)	India	Prospective, observational study, Kashmir Valley, India	2,245	51.0	33 ± 12	32 ± 11	33 ± 13	Simpson 2D	GE Vivid S5
Sengupta et al. [10] (2021)	India	INDEA	880	36.3	40 ± 12	39 ± 12	40 ± 13	Simpson 2D	GE Vivid E9 and E95
Yao et al. [19] (2015)	China	Prospective, nationwide, multicenter study	1,394	51.4	47 ± 16	48 ± 16	47 ± 16	Simpson 2D	Philips iE33 or the GE Vivid E9

WASE, World Alliance Society of Echocardiography; LOLIPOP, London Life Sciences Prospective Population; INDEA, Indian Normative Data of Echocardiography Analyzed

^a^Values are presented as mean ± standard deviation. ^b^Equipment not further specified

Most studies used Philips (iE33) or GE HealthCare (Vivid E9, E95, or S5) ultrasound platforms; one study also used Siemens equipment (model unspecified). LVEF was quantified using either the biplane Simpson method (2D) or volumetric analysis (3D echocardiography). The proportion of women in the included studies ranged from 36% [10] to 59% [11]. The mean age across included cohorts ranged from 27 years [12] to 54 years [13].

### Normal range of LVEF

**Fig. 1:**
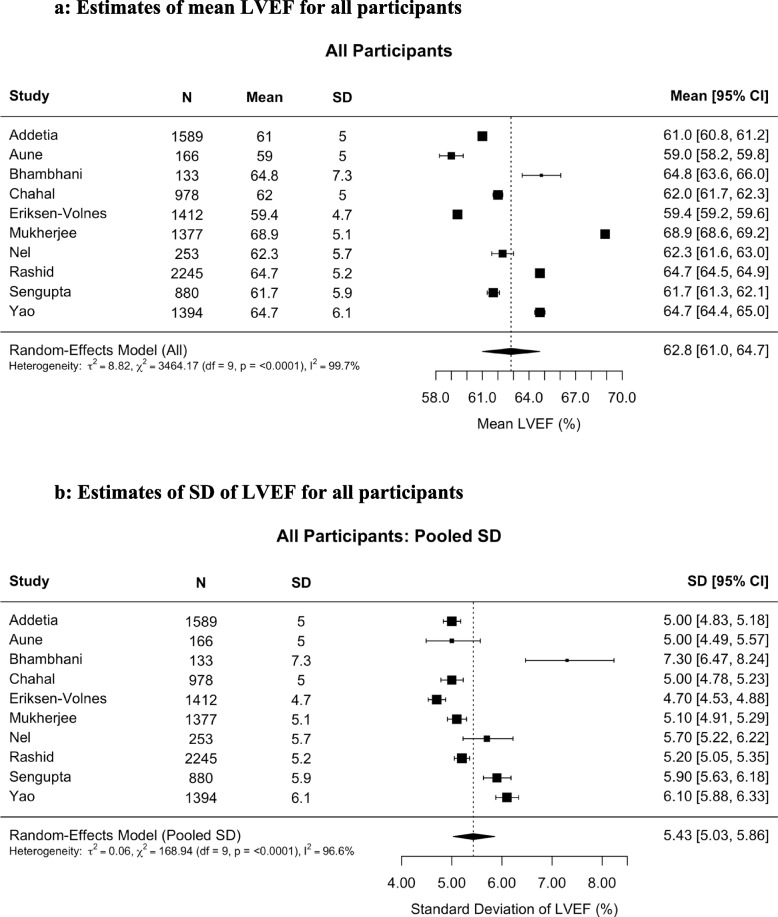
Estimates of (**A**) mean left ventricular ejection fraction (LVEF) and (**B**) pooled standard deviation (SD) of LVEF for all participants. For all participants (n = 10,427) the mean LVEF was 62.8% (95% confidence interval [CI], 61.0%–64.7%) calculated with a random-effects model. The pooled estimated SD was 5.43%. The lower and upper limits of normality were 52.21 and 73.49, respectively

**Fig. 2:**
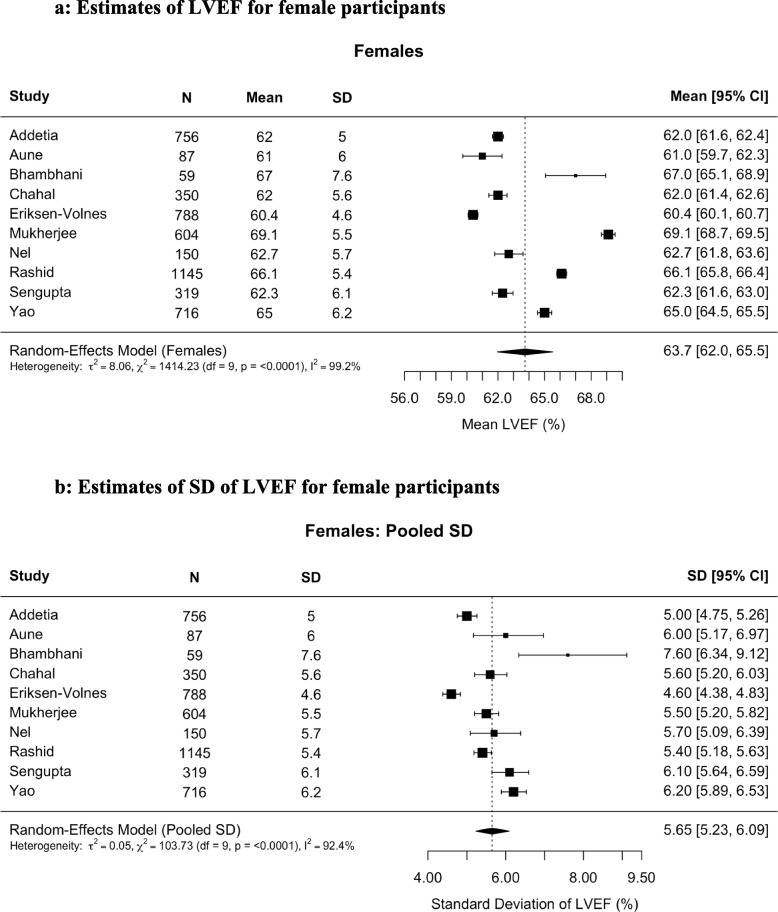
Estimates of (**A**) left ventricular ejection fraction (LVEF) and (**B**) pooled standard deviation (SD) of LVEF for female participants. The mean LVEF for female participants (n = 4,974) was 63.7% (95% confidence interval [CI], 62.0%–65.5%) calculated with a random-effects model. The pooled estimated SD was 5.65%. The lower and upper limits of normality were 52.68 and 74.81, respectively

**Fig. 3:**
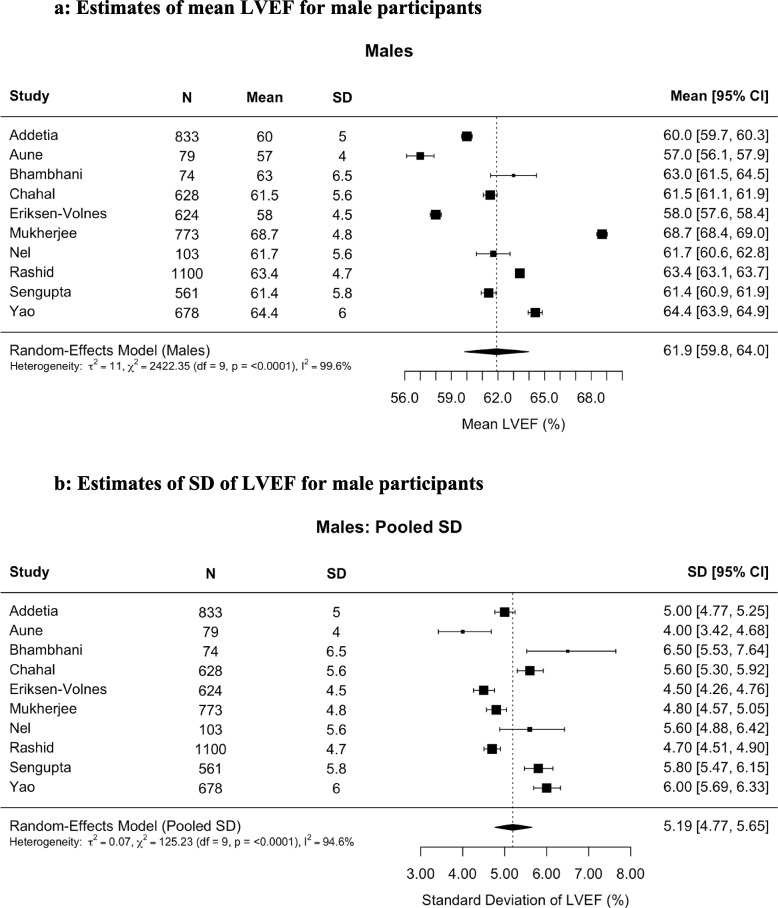
Estimates of (**A**) left ventricular ejection fraction (LVEF) and (**B**) pooled standard deviation (SD) of LVEF for male participants. For male participants (n = 5,453) the mean LVEF was 61.9% (95% confidence interval [CI], 59.8%–64.0%) calculated with a random-effects model. The pooled estimated SD was 5.19%. The lower and upper limits of normality were 51.74 and 72.09, respectively

**Fig. 4:**
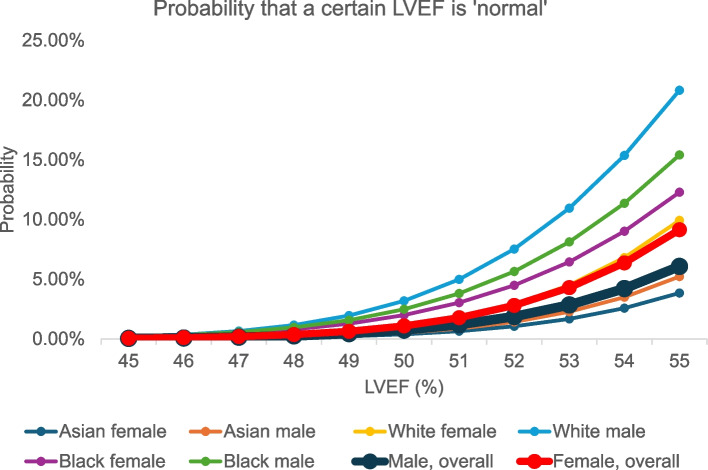
Probability that a certain left ventricular ejection fraction (LVEF) is “normal.”

Pooled estimates of normal mean LVEF are shown in Figs. 1, 2 and 3. Across all individuals, the mean LVEF was 62.8% (95% confidence interval [CI], 61.0%–64.7%) and pooled SD was 5.4% (95% CI, 5.0%–5.9%), with a lower reference limit of normality (LLN) of 52.2% and an upper limit of normality (ULN) of 73.5%. Women had a slightly higher mean LVEF (63.7%; 95% CI, 62.0%–65.5%) compared with men (61.9%; 95% CI, 59.8%–64.0%), with corresponding LLN and ULN at 52.7% and 74.8%, respectively, for women and 51.7% and 72.1%, respectively, for men. Given the normal distribution of LVEF in our healthy reference sample, fewer than 1% of women and only about 1% of men would be expected to have an LVEF below 50%. (Fig. 4, Supplementary Table 3).

### Age and normal LVEF

Six studies provided LVEF data stratified by age groups (Supplementary Table 4). Although interstudy heterogeneity was noted, there was no consistent trend suggesting significant variation in LVEF across different adult age strata. In meta-regression with age group (18–40, 41–65, > 65 years) as a categorical variable, there was no statistically significant difference in mean LVEF across age strata in women (overall P = 0.71) or men (overall P = 0.94).

### Race and normal LVEF

Subgroup analyses suggested modest differences in LVEF by race (Table 2) [10–19]. Asian populations demonstrated higher mean LVEF values compared with other groups (Asian women, 64.9% [95% CI, 62.9%–66.9%] and Asian men, 63.6% [95% CI, 61.6%–65.6%]; White women, 61.3% [95% CI, 60.5%–62.1%] and White men, 58.9% [95% CI, 57.2%–60.6%]; Black women, 61.5% [95% CI, 59.2%–63.9%] and Black men, 60.4% [95% CI, 57.8%–62.9%]). Corresponding LLN were also higher in the Asian group (53.8% for women and 53.2% for men) compared with other groups (LLN were approximately 51% for women and 50% for men). However, these estimates were subject to some variability, reflecting heterogeneity across the included Asian studies. The statistical likelihood that an LVEF < 50% would be normal for any sex and race was less than 4% (P < 0.04) for all groups (Fig. 4, Supplementary Table 5). In Asian populations, an LVEF < 54% in women and < 53% in men are less likely to be normal (P < 0.02).

**Table 2:** LVEF by self-reported race/ethnicity

Race/ethnicity	Women			Men		
	No. of subjects	LVEF (%)	std	No. of subjects	LVEF (%)	std
Asian population						
Bhambhani et al. [21] (India)	59	67	7.6	74	63	6.5
Mukherjee et al. [12] (India)	604	69.1	5.5	773	68.7	4.8
Rashid et al. [18] (India)	1,145	66.1	5.4	1,100	64.7	5.2
Sengupta et al. [10] (India)	319	62.3	6.1	561	61.4	5.8
Yao et al. [19] (China)	716	65	6.2	678	64.4	6
Chahal et al. [13] (UK, Indian Asian subsample)	189	62	5	290	62	5
Addetia et al. [14] (participating Asian countries)	293	62.9	4.6	333	61	4.5
Overall	3,325	64.9 (95% CI, 62.9–66.9)	5.6 (95% CI, 5.1–6.3)	3,809	63.6 (95% CI, 61.6–65.6)	5.3 (95% CI, 4.9–5.8)
Lower limit of normal		53.8			53.2	
Upper limit of normal		76.0			74.0	
τ^2^		7.0	0.1		7.1	0.1
I^2^		99.0	94.4		99.3	93.3
White population						
Aune et al. [15] (Norway)	87	61	6	79	57	4
Chahal et al. [13] (UK, European White subsample)	161	62	5	338	61	6
Eriksen-Volnes et al. [17] (Norway)	788	60.4	4.6	624	58	4.5
Addetia et al. [14] (White individuals)	277	61.8	4.3	291	59.4	4.8
Overall	1,313	61.3 (95% CI, 60.5–62.1)	4.9 (95% CI, 4.3–5.6)	1,332	58.9 (95% CI, 57.2.–60.5)	4.8 (95% CI, 4.1–5.7)
Lower limit of normal		51.7			49.4	
Upper limit of normal		70.9			68.3	
τ^2^		0.5	0.1		2.9	0.1
I^2^		85.7	88.4		97.2	93.7
Black population						
Nel et al. [11] (South Africa)	150	62.7	5.7	103	61.7	5.6
Addetia et al. [14] (Black individuals)	83	60.3	5.3	91	59.1	4.9
Overall	233	61.5 (95% CI, 59.2–63.9)	5.6 (95% CI, 5.1–6.1)	194	60.4 (95% CI, 57.8–62.9)	5.3 (95% CI, 4.6–6.0)
Lower limit of normal		50.6			50.1	
Upper limit of normal		72.4			70.7	
τ^2^		2.6	0.0		3.1	0.0
I^2^		90.4	0		91.6	41.4

LVEF, left ventricular ejection fraction; CI, confidence interval

### Normal LVEF in 3D vs. 2D echocardiography

Separate analyses of 3D versus 2D echocardiography cohorts are presented in Supplementary Figs. 2 and 3. These showed no systematic differences in LVEF and heterogeneity remained high in the two subgroups.

### Bias assessment

We performed a funnel plot to assess for bias in the published studies. This did not reveal any signs bias (Supplementary Fig. 4). Similarly, Egger regression test was nonsignificant (t = –0.37, df = 8, P = 0.72), indicating no evidence of funnel plot asymmetry or publication bias.

## Discussion

In this meta-analysis of over 10,000 healthy adults across diverse cohorts, we found that the lower limits of normality for LVEF were 52.7% for women and 51.7% for men, closely aligned with the thresholds proposed by the ASE/EACI guidelines [1]. Our results were derived from independent cohorts not included in the original ASE/EACI consensus, corroborating the validity of these guideline-based cutoffs.

To aid clinical interpretation, we provided a reference table for the statistical likelihood that an individual could have a “normal” LVEF even if below the defined cutoff points in our study (Fig. 4, Supplementary Tables 3, 5). Notably, an LVEF below 50% is highly likely to be abnormal, irrespective of age, sex, or race.

Our findings also support prior observations from the WASE study that individuals of Asian ancestry have slightly higher mean LVEF values, approximately 2 to 3 percentage points, compared with White and Black populations. Although our analysis included the WASE subsample, most Asian participants came from other cohorts, lending further credibility to the hypothesis that LVEF may be physiologically higher in this group. This has potential implications for diagnostic thresholds and therapeutic decision-making in Asian populations.

Sex-based differences were also evident, with women exhibiting slightly higher mean and lower limit LVEF values than men. These differences have been well documented and are consistent with current guideline recommendations. In contrast, we found no significant variation in LVEF by age group. Even among younger individuals, an LVEF < 50% appears unlikely to represent a normal variant. However, data for individuals aged ≥ 80 years were limited, and future studies are needed to define age-specific reference values in older adults.

The results of our analyses may also challenge the universal definition of heart failure with preserved versus reduced ejection fraction. An LVEF < 50% is currently used to define heart failure with reduced ejection fraction, which our data would be in support of, but could also suggest that several subgroups, for instance women and individuals of Asian origin, might more often have heart failure with reduced ejection fraction than currently recognized (e.g., if LVEF is 51%–53%). However, more research is needed before concluding that this may be the case (our study should be regarded as hypothesis generating only).

Although 2D echocardiography is more prone to measurement variability than 3D imaging, the pooled estimates in our analysis were consistent across modalities. Similarly, we observed no systematic differences in LVEF based on ultrasound machine manufacturer. Nevertheless, we acknowledge that methodological heterogeneity may contribute to residual uncertainty and should be considered when interpreting our results.

### Bias assessment

All included studies recruited apparently healthy adults, but the methods of participant selection varied. As with most studies involving voluntary participation, some degree of selection bias is possible, as individuals who enroll in health studies may be healthier than the general population. However, given that our objective was to define normal reference values, this selection is consistent with the study goal. Moreover, the likelihood of publication bias is considered minimal, as the studies were descriptive and not hypothesis driven.

### Strengths and limitations

This study provides comprehensive, demographically stratified LVEF reference values using data from population-based cohorts. It includes representation from multiple continents and imaging platforms. However, most included participants were of European or Asian ancestry. Data from individuals of Hispanic and Black races were limited, restricting generalizability to those populations. Further studies are therefore warranted to establish normal LVEF ranges in these underrepresented groups. Additionally, both 2D and 3D imaging modalities were included, and echocardiographic methods were not standardized across studies. We included the WASE 3D cohort (n = 1,589) but not the 2D WASE cohort (n = 2,008) because the 2D publication reports percentile-based reference limits only and does not provide mean ± SD for LVEF and individual-level data were unavailable. We acknowledge that excluding this core-lab–adjudicated dataset may reduce comparability with 2D-focused practice; however, our main conclusions were unchanged in analyses across other large cohorts and further supported by similar conclusions as drawn in the WASE 2D study [6]. The high I^2^ and τ^2^ values need to be emphasized as a potentially significant limitation. For instance, the different cohorts used different exclusion criteria to define “healthy,” and especially the body mass index (BMI) cutoff varied among studies. Although prior studies have suggested that LVEF is not influenced by BMI [20–23], it cannot be fully excluded that this influenced our results. Differences in imaging acquisition protocols might also have contributed to the heterogeneity. That being said, high I^2^ values do not always indicate a large variance in effects between studies or reflect fundamentally incompatible populations [24]. In our meta-analysis, the mean LVEFs were distributed across the 60% to 69% range without disconcordant outliers, and the high I^2^ values may to some extent reflect small within-study standard errors on a large, combined sample size. The between-study variance was τ^2^ = 7–8, corresponding to a SD of the true effects (τ) of approximately 2.7 percentage points, which appeared to be partly driven by race (as stratified analyses showed less variation, except for individuals of Asian descent). Overall, however, the results should be interpreted cautiously considering the observed heterogeneity and the potential influence of population selection criteria and methodological differences.

## Conclusions

In this meta-analysis of healthy adults, the lower limit of normal LVEF was 53% in women and 52% in men. Individuals of Asian origin had slightly higher LVEF values, with corresponding lower limit of normal of approximately 54% for women and 53% for men. An LVEF < 50% is highly likely to reflect abnormal systolic function, regardless of age, sex, or race. Future research is needed to define normative LVEF in specific subgroups, including Hispanic populations and individuals ≥ 80 years of age.

## Supplementary Information

Supplementary Material 1: Table S1. Search strings. Table S2. Exclusion criteria in included articles. Table S3. Statistical likelihood that certain values of LVEF could be “normal”. Table S4. Values by age groups and sex in the subset of available studies. Table S5. Likelihood of an LVEF being normal in sex- and race-specific subgroups. Fig. S1. Flowchart. Fig. S2. Meta-analysis of studies using 3D echocardiography. Fig. S3. Meta-analysis of studies using 3D echocardiography. Fig. S4. Funnel plot of included studies.

## Data Availability

No datasets were generated or analysed during the current study.

## Abbreviations

ASE: American Society of Echocardiography
BMI: Body mass index
CI: Confidence interval
EACI: European Association of Cardiovascular Imaging
LLN: Lower reference limit of normality
LVEF: Left ventricular ejection fraction
PRISMA: Preferred Reporting Items for Systematic reviews and Meta-Analyses
SD: Standard deviation
ULN: Upper limit of normality
WASE: World Alliance Society of Echocardiography
