# Supplementary material for: What is a normal left ventricular ejection fraction in healthy adults? A meta-analysis of population-based echocardiographic studies
Source: J Cardiovasc Imaging. 2026 Jan 22;34:2. doi: 10.1186/s44348-025-00063-4 (PMC12828944; doi:10.1186/s44348-025-00063-4)
Supplement: Supplementary file 1 — Supplementary Material 1: Table S1. Search strings. Table S2. Exclusion criteria in included articles. Table S3. Statistical likelihood that certain values of LVEF could be “normal”. Table S4. Values by age groups and sex in the subset of available studies. Table S5. Likelihood of an LVEF being normal in sex- and race-specific subgroups. Fig. S1. Flowchart. Fig. S2. Meta-analysis of studies using 3D echocardiography. Fig. S3. Meta-analysis of studies using 3D echocardiography. Fig. S4. Funnel plot of included studies. [file 44348_2025_63_MOESM1_ESM.docx]

# Supplementary materials

#### **Supplementary Fig. 1.** Flowchart


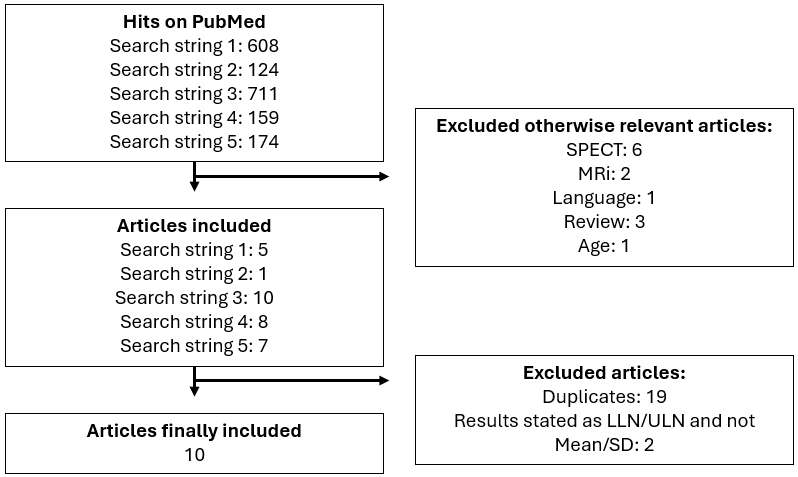


#### **Supplementary Fig. 2. Meta-analysis of studies using 3D echocardiography.**

**A. Mean LVEF**


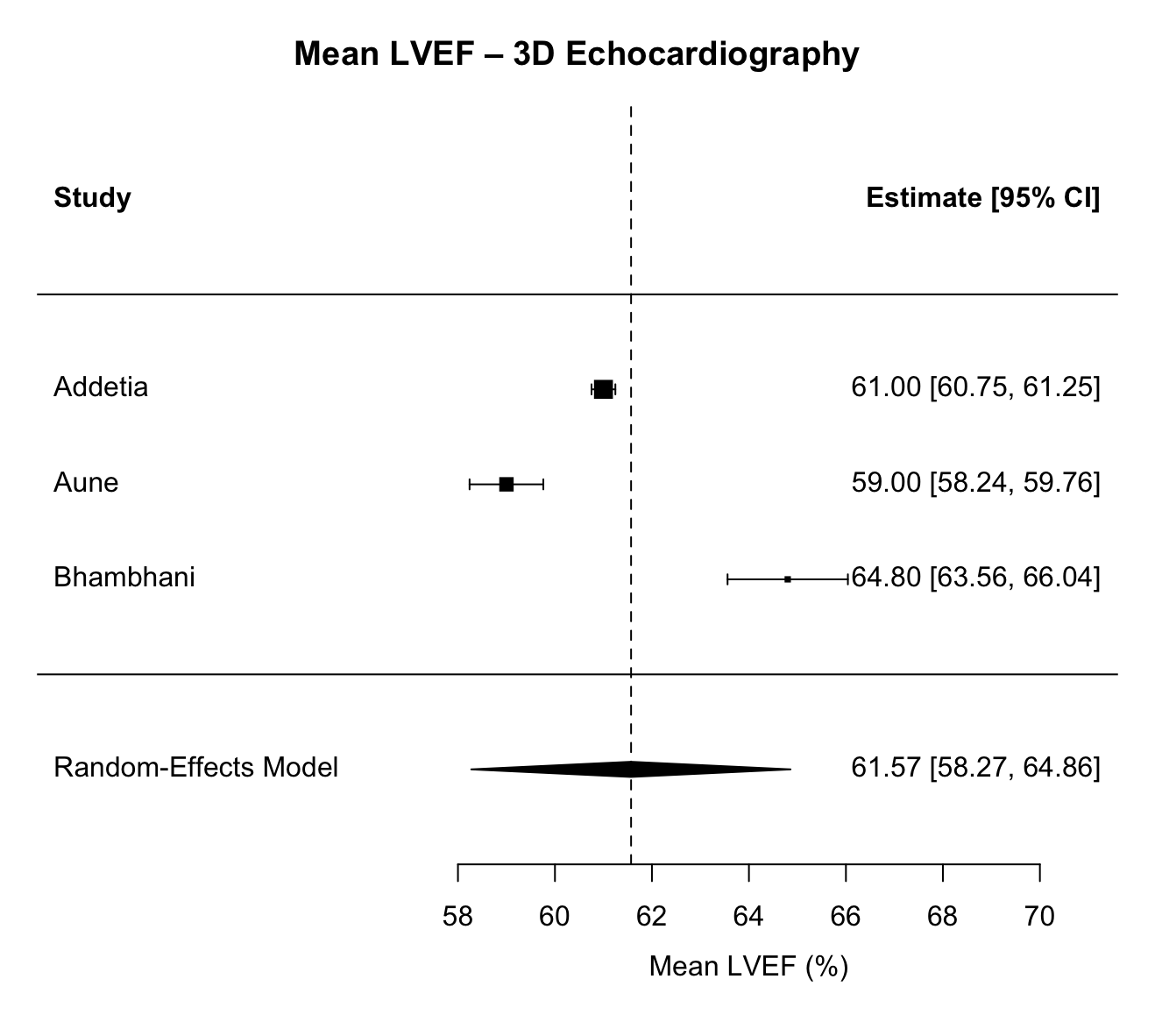


τ² value 8.28, I^2^ 98.6%.

#### **Supplementary Figure 2 – meta-analysis of studies using 3D echocardiography.**

#### **B. Standard deviation LVEF**

***
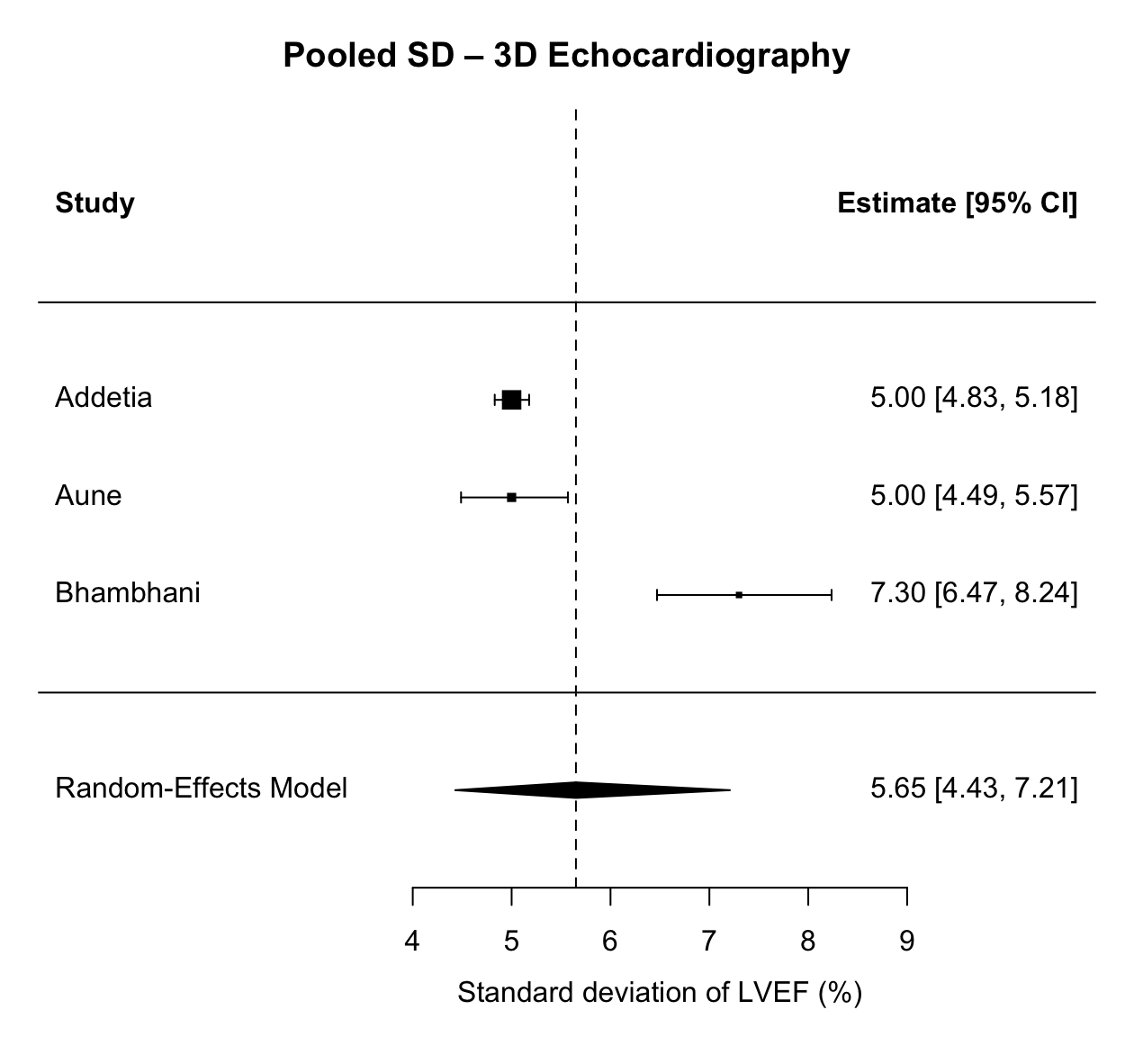
***

τ² value 0.18, I^2^ 95.8%.

#### **Supplementary Figure 3 – meta-analysis of studies using 2D echocardiography.**

**A. Mean LVEF**

***
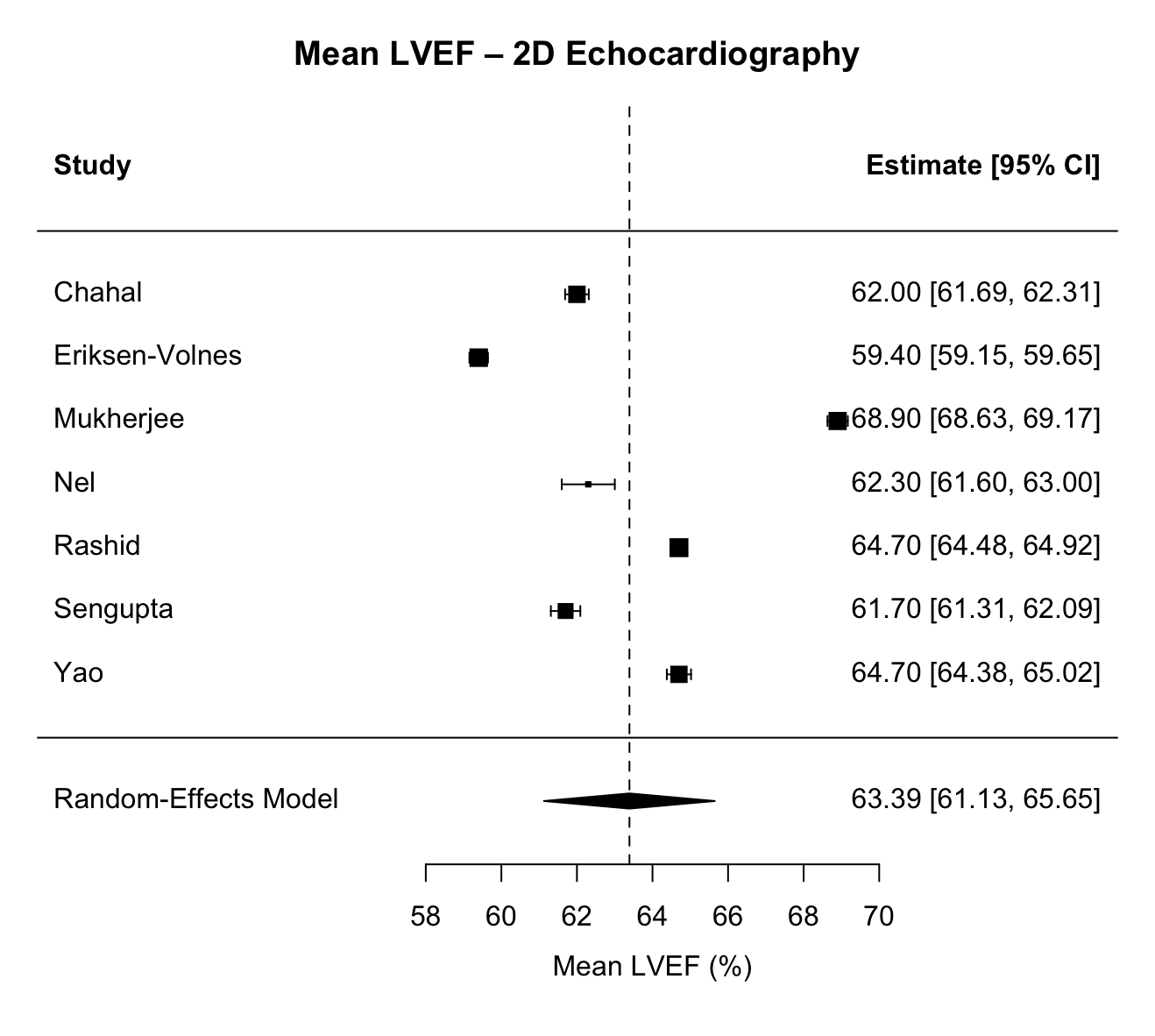
***

τ² value 9.28, I^2^ 99.8%.

#### **Supplementary Figure 3 – meta-analysis of studies using 2D echocardiography.**

**B. Standard deviation LVEF**

τ² value 0.035, I^2^ 95.4%. ***
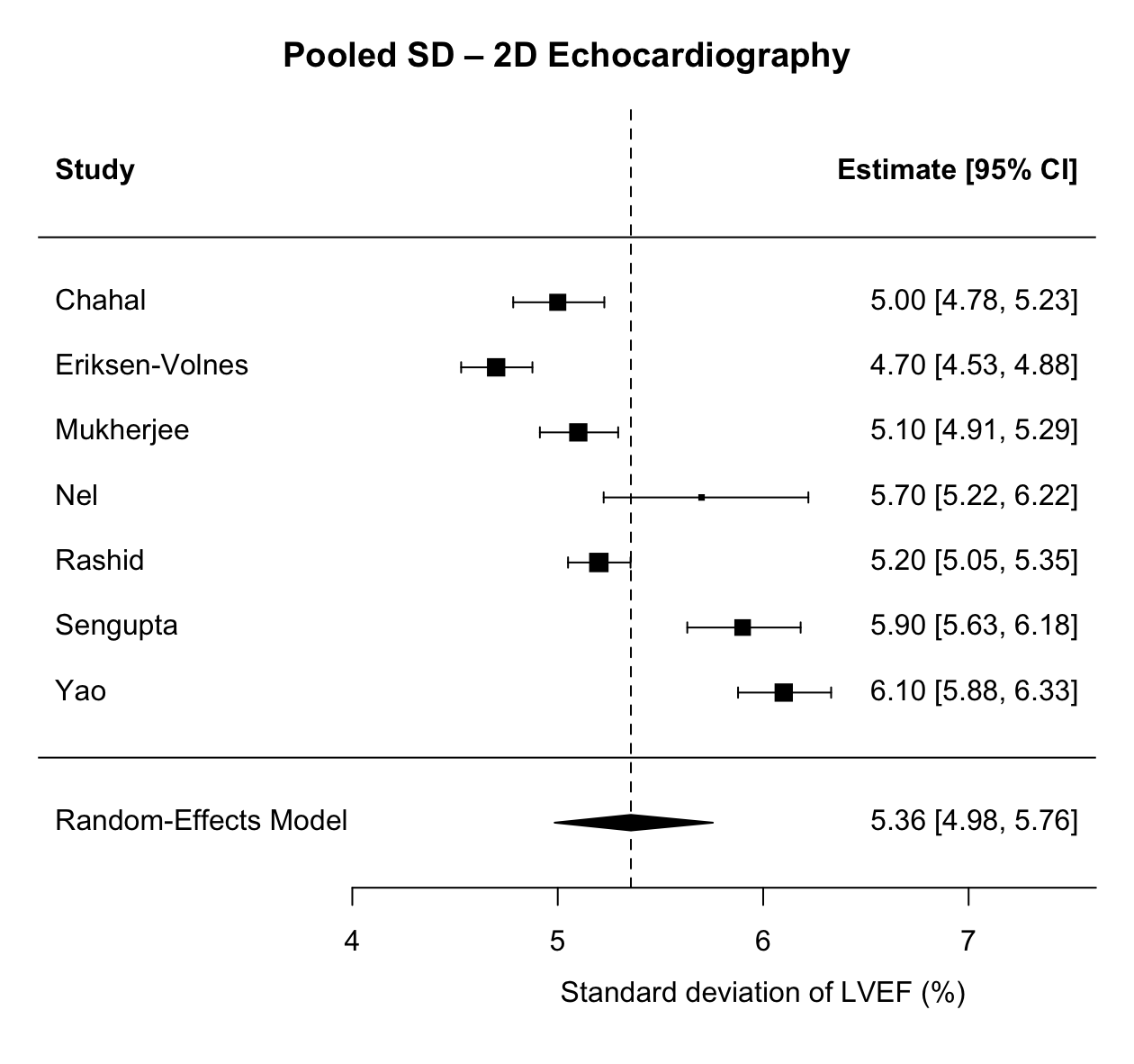
***

**Supplementary Figure 4 – Funnel plot of included studies**


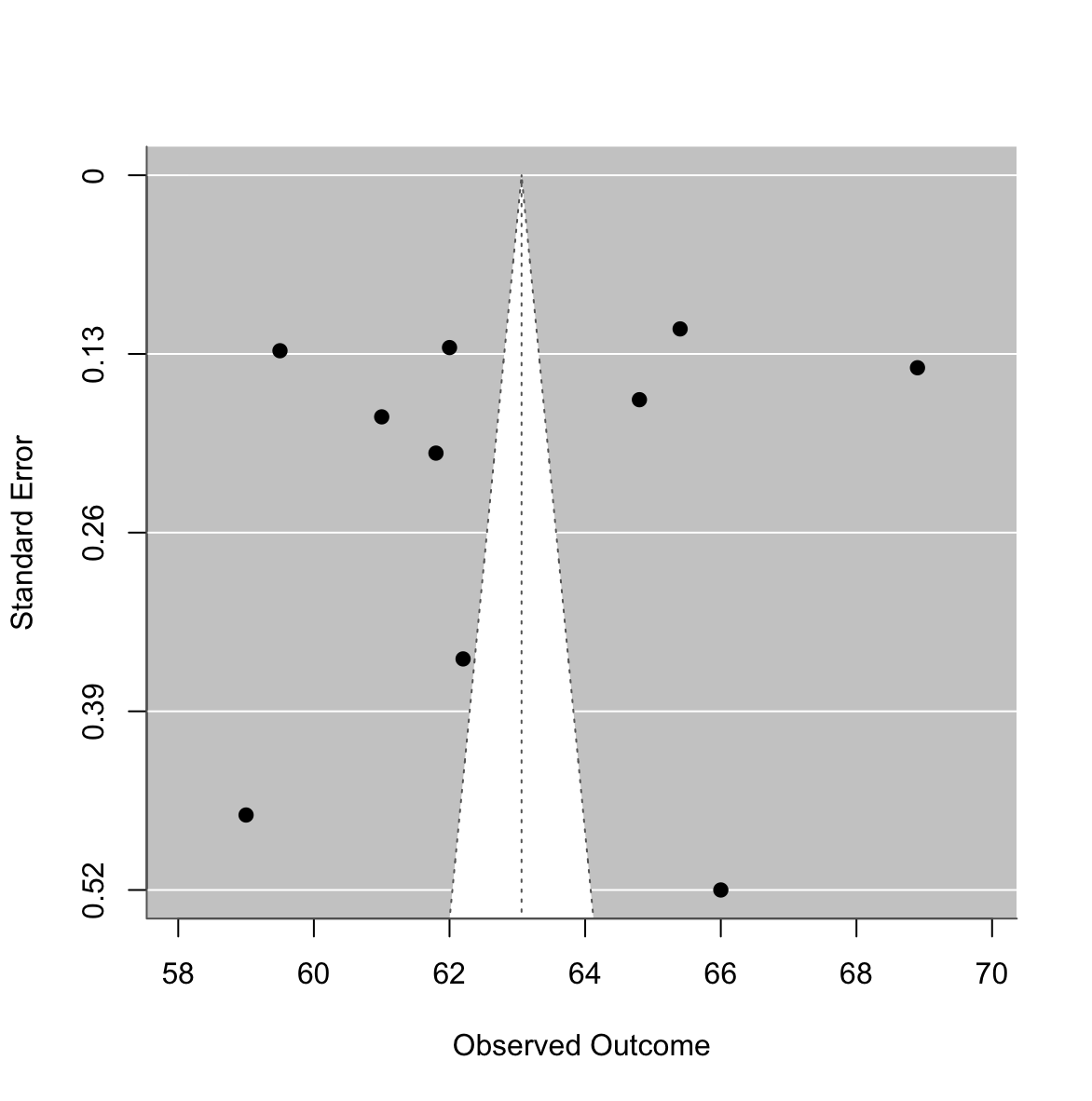


#### **Supplementary Table 1. Search strings**

| Search string 1 | (“left ventricular ejection fraction” OR “LVEF”) AND ("sex" OR “gender” OR “male” OR “female” OR “men” OR “women”) AND ("mean value" OR “normal”) AND (“population based” OR “population-based” OR “cohort” OR “community”) |
| --- | --- |
| Search string 2 | (“left ventricular ejection fraction” OR “LVEF”) AND ("sex-specific”) |
| Search string 3 | (“left ventricular ejection fraction” OR “LVEF”) AND ("sex” OR “gender”) AND (“normal”) |
| Search string 4 | ("left ventricular ejection fraction" OR "LVEF") AND ("echo*" OR "magnetic") AND ("normal" OR "population-based" OR "cohort") NOT ("heart failure" OR "dysfunction" OR "cancer" OR "cardiomyopathy" OR "pulmonary") AND (“healthy”) |
| Search string 5 | ("left ventricular ejection fraction" OR "LVEF") AND ("normal value*") |

#### **Supplementary Table 2. Exclusion criteria in included articles**

| **Exclusion criteria** | **Articles** | **n** |
| --- | --- | --- |
| Age $<$ 18 | Addetia^14)^, Aune^15)^, Bhambhani^16)^, Chahal^13)^, Eriksen-Volnes^17)^, Mukherjee^12)^, Nel^11)^, Rashid^18)^, Sengupta^10)^, Yao^19)^ | 10 |
| Cardiac/coronary disease / abnormalities including infarcts including treatment of it, HF | Addetia^14)^, Aune^15)^, Bhambhani^16)^, Chahal^13)^, Eriksen- Volnes^17)^, Mukherjee^12)^, Nel^11)^, Rashid^18)^, Sengupta^10)^, Yao^19)^ | 10 |
| History or current use of antihypertensives / hypertension (> 160 / 90 mmHg) > 140 / 90 mmHg, 140 / 85 mmHg | Addetia^14)^, Aune^15)^, Bhambhani^16)^, Chahal^13)^, Eriksen-Volnes^17)^, Mukherjee^12)^, Nel^11)^, Rashid^18)^, Sengupta^10)^, Yao^19)^ | 10 |
| Diabetes type 1 / 2 or impaired fasting blood glucose ($\geq$ 100 mg/dL), > 7 mmol/L | Addetia^14)^, Aune^15)^, Chahal^13)^, Eriksen-Volnes^17)^, Mukherjee^12)^, Nel^11)^, Rashid^18)^, Sengupta^10)^, Yao^19)^ | 9 |
| Incomplete echocardiography / poor image quality | Addetia^14)^, Aune^15)^, Nel^11)^, Sengupta^10)^, Yao^19)^ | 5 |
| Pregnancy or lactating women | Addetia^14)^, Mukherjee^12)^, Sengupta^10)^, Yao^19)^ | 4 |
| Dyslipidemia | Addetia^14)^, Mukherjee^12)^, Rashid^18)^, Yao^19)^ | 4 |
| Pulmonary disease | Addetia^14)^, Eriksen-Volnes^17)^, Mukherjee^12)^, Yao^19)^ | 4 |
| Treatment for or current arrythmia including AF | Aune^15)^, Eriksen-Volnes^17)^, Rashid^18)^, Sengupta^10)^, | 4 |
| Obesity (BMI >30, > 28) | Mukherjee^12)^, Rashid^18)^, Sengupta^10)^, Yao^19)^ | 4 |
| Renal disease / renal insufficiency / dysfunction | Addetia^14)^, Mukherjee^12)^, Rashid^18)^ | 3 |
| Competitive / professional athlete or long-term physical training | Addetia^14)^, Mukherjee^12)^, Yao^19)^ | 3 |
| History of or current alcoholism | Addetia^14)^, Mukherjee^12)^, Yao^19)^ | 3 |
| Malignant disease | Aune^15)^, Eriksen-Volnes^17)^, Sengupta^10)^ | 3 |
| Angina pectoris | Eriksen-Volnes^17)^, Mukherjee^12)^ | 2 |
| Abnormal 12-lead ECG | Nel^11)^, Yao^19)^ | 2 |
| Prior stroke | Nel^11)^, Yao^19)^ | 2 |
| LBBB | Aune^15)^ | 1 |
| Indian Asian ancestry | Chahal^13)^ | 1 |
| European ancestry | Chahal^13)^ | 1 |
| Thyroid disease | Mukherjee^12)^ | 1 |
| Treatment with corticosteroids | Mukherjee^12)^ | 1 |
| Any chronic medical condition | Nel^11)^ | 1 |
| Any chronic medication | Nel^11)^ | 1 |
| HR > 100 BPM | Sengupta^10)^ | 1 |
| Any endocrine disease | Yao^19)^ | 1 |
| Anemia | Yao^19)^ | 1 |
| Connective tissue disease | Yao^19)^ | 1 |
| Abnormal hepatic function (serum alanine / aspartate aminotransferase > 2x upper limit) | Yao^19)^ | 1 |

#### **Supplementary Table 3: Statistical likelihood that certain values of LVEF could be “normal”**

| **LVEF (%)** | **All**  **Mean = 62.8, SD = 5.43** | |  | **Female**  **Mean = 63.7, SD = 5.65** | | | **Male**  **Mean = 61.9, SD = 5.19** | | |
| --- | --- | --- | --- | --- | --- | --- | --- | --- | --- |
|  | **Z-score** | **Prob (≤ X)** | **p-value** | **Z-score** | **Prob (≤ X)** | **p-value** | **Z-score** | **Prob (≤ X)** | **p-value** |
| **55** | -1.45 | 7.41% | 0.0741 | -1.55 | 6.08% | 0.0608 | -1.33 | 9.15% | 0.0915 |
| **54** | -1.63 | 5.15% | 0.0515 | -1.73 | 4.22% | 0.0422 | -1.52 | 6.37% | 0.0637 |
| **53** | -1.81 | 3.48% | 0.0348 | -1.90 | 2.85% | 0.0285 | -1.72 | 4.30% | 0.0430 |
| **52** | -2.00 | 2.28% | 0.0228 | -2.08 | 1.88% | 0.0188 | -1.91 | 2.81% | 0.0281 |
| **51** | -2.18 | 1.45% | 0.0145 | -2.26 | 1.20% | 0.0120 | -2.10 | 1.78% | 0.0178 |
| **50** | -2.37 | 0.90% | 0.0090 | -2.43 | 0.75% | 0.0075 | -2.29 | 1.09% | 0.0109 |
| **49** | -2.55 | 0.54% | 0.0054 | -2.61 | 0.45% | 0.0045 | -2.49 | 0.64% | 0.0064 |
| **48** | -2.74 | 0.31% | 0.0031 | -2.79 | 0.26% | 0.0026 | -2.68 | 0.37% | 0.0037 |
| **47** | -2.92 | 0.18% | 0.0018 | -2.97 | 0.15% | 0.0015 | -2.87 | 0.20% | 0.0020 |
| **46** | -3.10 | 0.10% | 0.0010 | -3.14 | 0.08% | 0.0008 | -3.06 | 0.11% | 0.0011 |
| **45** | -3.29 | 0.05% | 0.0005 | -3.32 | 0.05% | 0.0005 | -3.26 | 0.06% | 0.0006 |

#### **Supplementary Table 4: Values by age groups and sex in the subset of available studies**

|  | **Female** | | | **Male** | | |
| --- | --- | --- | --- | --- | --- | --- |
| **Age** | **18-40** | **41-65** | **>65** | **18-40** | **41-65** | **>65** |
| Addetia et al, N | 309 | 275 | 172 | 347 | 291 | 195 |
| LVEF mean, % | 61.8 | 62.3 | 62.7 | 59.5 | 60.3 | 61.2 |
| Std | 4.3 | 4.4 | 5.3 | 4.4 | 4.7 | 5.1 |
| Bhambhani et al., N | 30 | 29 |  | 43 | 31 |  |
| LVEF mean, % | 65.1 | 69.1 |  | 63.9 | 61.7 |  |
| Std | 6.3 | 8.3 |  | 6.5 | 6.4 |  |
| Eriksen-Volnes et al., N | 64 | 367 | 367 | 49 | 284 | 291 |
| LVEF mean, % | 60 | 61 | 60 | 58 | 60 | 60 |
| Std | 4 | 5 | 5 | 6 | 5 | 5 |
| Mukherjee et al., N | 604 |  |  | 773 |  |  |
| LVEF mean, % | 69.1 |  |  | 68.7 |  |  |
| std | 5.5 |  |  | 4.8 |  |  |
| Rashid et al, N | 905 | 240 |  | 750 | 350 |  |
| LVEF mean, % | 66.5 | 66.1 |  | 63.2 | 63.7 |  |
| std | 5.2 | 5.6 |  | 5.1 | 5.3 |  |
| Yao et al., N | 255 | 276 | 185 | 246 | 244 | 188 |
| LVEF mean, % | 64.7 | 64.7 | 65.8 | 63 | 64.7 | 65.7 |
| std | 6.3 | 6.2 | 6 | 5.8 | 6 | 6 |
| **Summary** |  |  |  |  |  |  |
| **Total N across studies** | **2,167** | **1,187** | **724** | **1,435** | **1,200** | **674** |
| **LVEF mean, % (95% CI)** | **64.5 (61.9, 67.2)** | **64.4 (61.8, 67.1)** | **62.8 (59.5, 66.1)** | **62.7 (59.7, 65.8)** | **62.1 (60.2, 64.0)** | **62.3 (58.9, 65.7)** |
| **Std (95% CI)** | **5.2 (4.5, 6.0)** | **5.6 (4.7, 6.8)** | **5.4 (4.8, 6.0)** | **5.3 (4.7, 5.9)** | **5.3 (4.8, 5.9)** | **5.3 (4.8, 6.0)** |
| **Upper and lower limit of normal LVEF, %** | **54.4, 74.7** | **53.4, 75.5** | **52.2, 73.4** | **52.4, 73.1** | **51.6, 72.5** | **51.8, 72.7** |
| $\tau^{2}$ (mean) | 10.6 | 8.5 | 8.3 | 13.8 | 4.4 | 8.9 |
| $\tau^{2}$ (SD) | 0.1 | 0.2 | 0.0 | 0.1 | 0.0 | 0.0 |
| $I^{2}$ (mean) | 99.2% | 98.6% | 98.4% | 99.4% | 97.3% | 98.6% |
| $I^{2}$ (SD) | 94.6% | 94.9% | 74.7% | 91.0% | 81.2% | 76.5% |

Footnote: Age groups are approximate as the split differed slightly in the various studies.

#### **Supplementary Table 5: Likelihood of an LVEF being normal in sex- and race-specific subgroups.**

|  | **Asian female** | | | **Asian male** | | |
| --- | --- | --- | --- | --- | --- | --- |
| **LVEF (%)** | **Z-score** | **Prob (≤ X)** | **p-value** | **Z-score** | **Prob (≤ X)** | **p-value** |
| **55** | -1.77 | 3.85% | 0.0385 | -1.62 | 5.23% | 0.0523 |
| **54** | -1.95 | 2.58% | 0.0258 | -1.81 | 3.50% | 0.035 |
| **53** | -2.13 | 1.68% | 0.0168 | -2 | 2.28% | 0.0228 |
| **52** | -2.3 | 1.06% | 0.0106 | -2.19 | 1.43% | 0.0143 |
| **51** | -2.48 | 0.65% | 0.0065 | -2.38 | 0.87% | 0.0087 |
| **50** | -2.66 | 0.39% | 0.0039 | -2.57 | 0.51% | 0.0051 |
| **49** | -2.84 | 0.23% | 0.0023 | -2.75 | 0.29% | 0.0029 |
| **48** | -3.02 | 0.13% | 0.0013 | -2.94 | 0.16% | 0.0016 |
| **47** | -3.2 | 0.07% | 0.0007 | -3.13 | 0.09% | 0.0009 |
| **46** | -3.38 | 0.04% | 0.0004 | -3.32 | 0.04% | 0.0004 |
| **45** | -3.55 | 0.02% | 0.0002 | -3.51 | 0.02% | 0.0002 |
|  | **White female** | | | **White male** | | |
| **LVEF (%)** | **Z-score** | **Prob (≤ X)** | **p-value** | **Z-score** | **Prob (≤ X)** | **p-value** |
| **55** | -1.29 | 9.93% | 0.0993 | -0.81 | 20.83% | 0.2083 |
| **54** | -1.49 | 6.81% | 0.0681 | -1.02 | 15.37% | 0.1537 |
| **53** | -1.69 | 4.51% | 0.0451 | -1.23 | 10.95% | 0.1095 |
| **52** | -1.9 | 2.89% | 0.0289 | -1.44 | 7.53% | 0.0753 |
| **51** | -2.1 | 1.78% | 0.0178 | -1.65 | 4.99% | 0.0499 |
| **50** | -2.31 | 1.06% | 0.0106 | -1.85 | 3.19% | 0.0319 |
| **49** | -2.51 | 0.60% | 0.006 | -2.06 | 1.96% | 0.0196 |
| **48** | -2.71 | 0.33% | 0.0033 | -2.27 | 1.16% | 0.0116 |
| **47** | -2.92 | 0.18% | 0.0018 | -2.48 | 0.66% | 0.0066 |
| **46** | -3.12 | 0.09% | 0.0009 | -2.69 | 0.36% | 0.0036 |
| **45** | -3.33 | 0.04% | 0.0004 | -2.9 | 0.19% | 0.0019 |
|  | **Black female** | | | **Black male** | | |
| **LVEF (%)** | **Z-score** | **Prob (≤ X)** | **p-value** | **Z-score** | **Prob (≤ X)** | **p-value** |
| **55** | -1.16 | 12.29% | 0.1229 | -1.02 | 15.41% | 0.1541 |
| **54** | -1.34 | 9.02% | 0.0902 | -1.21 | 11.36% | 0.1136 |
| **53** | -1.52 | 6.45% | 0.0645 | -1.4 | 8.13% | 0.0813 |
| **52** | -1.7 | 4.49% | 0.0449 | -1.58 | 5.65% | 0.0565 |
| **51** | -1.88 | 3.04% | 0.0304 | -1.77 | 3.81% | 0.0381 |
| **50** | -2.05 | 2.00% | 0.02 | -1.96 | 2.49% | 0.0249 |
| **49** | -2.23 | 1.28% | 0.0128 | -2.15 | 1.57% | 0.0157 |
| **48** | -2.41 | 0.80% | 0.008 | -2.34 | 0.97% | 0.0097 |
| **47** | -2.59 | 0.48% | 0.0048 | -2.53 | 0.57% | 0.0057 |
| **46** | -2.77 | 0.28% | 0.0028 | -2.72 | 0.33% | 0.0033 |
| **45** | -2.95 | 0.16% | 0.0016 | -2.91 | 0.18% | 0.0018 |
